# Effect of high intake of cod or salmon on serum total neopterin concentration: a randomised clinical trial

**DOI:** 10.1007/s00394-021-02497-0

**Published:** 2021-02-12

**Authors:** Anita Helland, Marianne Bratlie, Ingrid V. Hagen, Øivind Midttun, Harald Sveier, Gunnar Mellgren, Per Magne Ueland, Oddrun A. Gudbrandsen

**Affiliations:** 1 Department of Clinical Medicine, University of Bergen, Haukeland University Hospital, 5021 Bergen, Norway; 2 Bevital AS, Jonas Lies veg 87, 5021 Bergen, Norway; 3 Lerøy Seafood Group ASA, PO Box 7600, 5020 Bergen, Norway; 4 Department of Clinical Science, University of Bergen, 5021 Bergen, Norway; 5 Hormone Laboratory, Department of Medical Biochemistry and Pharmacology, Haukeland University Hospital, 5021 Bergen, Norway

**Keywords:** Atlantic cod, Atlantic salmon, Neopterin, Immune activation, Fish intake

## Abstract

**Purpose:**

Primarily, to investigate the effect of high intake of cod (lean fish) or salmon (fatty fish) on serum concentration of total neopterin, a marker of cellular immune activation that is associated with cardiovascular disease. Second, to investigate effects of high cod/salmon intake on antioxidant vitamins and elements essential for activity of antioxidant enzymes.

**Methods:**

In this randomised clinical trial, 63 participants with overweight/obesity consumed 750 g/week of either Atlantic cod (*N* = 22) or Atlantic salmon (*N* = 22) or were instructed to continue their normal eating habits but avoid fish intake (Control group, *N* = 19) for 8 weeks. Food intake was recorded, and fasting serum were collected at baseline and endpoint.

**Results:**

Serum total neopterin concentration was reduced in the Cod group (median change − 2.65 (25th, 75th percentiles − 3.68, − 0.45) nmol/l, *P* = 0.018) but not in the Salmon group (median change 0.00 (25th, 75th percentiles − 4.15, 3.05) nmol/l, *P* = 0.59) when compared with the Control group after 8 weeks. The estimated daily intake of selenium, iron, magnesium and zinc were similar between all groups. Increased serum concentration of selenium was observed only after cod intake when compared to the Control group (*P* = 0.017). Changes in serum concentrations of copper, iron, magnesium, all-*trans* retinol, α-tocopherol and γ-tocopherol were similar between the groups.

**Conclusion:**

A high intake of cod, but not of salmon, lowered serum total neopterin concentration when compared to the Control group.

**Clinical trial registration:**

This trial was registered at clinicaltrials.gov as NCT02350595

## Introduction

Chronic low-grade inflammation is a common feature in obesity, and is suggested to be an important element in the development of obesity-related co-morbidities including insulin resistance and diabetes mellitus type 2 [1–3]. The cytokine interferon-γ (IFNγ), produced by activated T cells, induces the metabolism of 7,8-dihydroneopterin from guanosine triphosphate in monocyte-derived macrophages during immune activation [4]. 7,8-dihydroneopterin is a potent radical scavenger and antioxidant, and its conversion to neopterin through non-enzymatic oxidation depends on the oxidative status of the individual; therefore the combined measurement of 7,8-dihydroneopterin and neopterin provide a sensitive measure for cellular immune activation [4]. IFNγ is also an important trigger for the production of reactive oxygen species in macrophages [5], thus neopterin may also be an indicator of oxidative stress due to immune activation [6], and circulating neopterin concentration has been shown to be inversely correlated with the concentrations of antioxidant enzyme cofactor selenium [7] and antioxidant vitamins ascorbic acid and α-tocopherol [8] in the circulation.

Elevated neopterin concentration, measured either as neopterin or as the sum of 7,8-dihydroneopterin and neopterin (total neopterin), in circulation is reported in patients with chronic peripheral arterial disease [9]. Since IFNγ quickly binds to soluble receptors or target structures after secretion to circulation, its half-life is short and the IFNγ concentration is difficult to measure, and circulating neopterin is a more reliable measure of immune activation [10]. Circulating neopterin is also associated with adverse prognosis in patients with stable angina pectoris [11] and increased risk of inpatient hospital diagnosis of atrial fibrillation [12], and is an independent predictor of all-cause and cardiovascular mortality in individuals with or without stable coronary artery disease [13]. High BMI, adiposity and elevated plasma glucose are also associated with higher circulating concentration of neopterin in healthy adults, suggesting that moderate immune stimulation plays a role in the development of insulin resistance [14, 15]. It is of great importance that a reduction in neopterin concentration of only 1–2 nmol/l, especially in the lower concentration area, has been associated with lower risk for acute coronary events in elderly adults without pre-existing coronary heart disease [16].

A significant negative correlation between intake of total fish and lean fish, but not of fatty fish, with plasma neopterin concentration in patients with coronary artery disease has been reported [17]. Fish consumption has been associated with lower circulating concentrations of several inflammatory markers in healthy adults; however, several studies report of no or a negative correlation of fish intake with inflammatory markers in clinical trials [18]. Serum neopterin concentration has been shown to be inversely correlated with antioxidant enzyme cofactor selenium and antioxidant vitamins in circulation [7, 8], but little is known about any relations of serum neopterin with copper, iron and magnesium, which also are essential for the activity of antioxidant enzymes. Based on the current available knowledge that individuals with overweight/obesity have elevated circulating neopterin concentration and that intake of total and lean fish are associated with lower neopterin concentration, we wanted to investigate the effect of high cod or salmon intake on serum neopterin in a group of adults with overweight/obesity.

We have previously demonstrated that a high salmon intake (750 g/week) for 8 weeks improved post-prandial glucose regulation in study participants with overweight/obesity, whereas high cod intake (750 g/week) did not affect glucose regulation [19]. In the same study, we also found that 750 g/week of salmon was not sufficient to prevent a decrease in serum 25-hydroxyvitamin D3 in autumn in South-Western Norway at 60° north latitude [20] but did affect gut microbiota profile [21]. In the present study, we wanted to further explore the biological materials, i.e. serum and urine samples, from this randomised clinical trial, with the main aim to gain insight into if a high intake of cod or salmon would affect serum total neopterin concentration. The secondary aims of this study were to investigate effects of high intake of cod or salmon on estimated dietary intakes of iron, magnesium, selenium and zinc and on serum concentrations of copper, iron, magnesium, selenium, all-*trans* retinol α-tocopherol and γ-tocopherol. Our hypothesis was that a high intake of cod would reduce serum total neopterin concentration.

## Methods

### Participants, study setting and ethics

The study design, the description of study participants, the study setting and the protocol for study visits have been published in detail previously [19]. In brief, the study population consisted of adults with overweight or obesity, and all participants were of Norwegian ethnic origin (Caucasian) living in the Bergen area in South-Western Norway. Inclusion criteria were; BMI ≥ 27 kg/m^2^, fasting blood glucose ≤ 7·0 mmol/l and age 18–69 years. Exclusion criteria were high habitual fish/seafood intake (> 500 g/week), pregnancy, incompatibility with fish consumption (allergies, intolerance and/or dislike), diagnosed diabetes mellitus, heart disease or gastrointestinal diseases, use of medications affecting lipid metabolism or glucose homoeostasis, use of anti-inflammatory medications, use of supplements containing n-3 PUFAs, intentional weight loss and large fluctuation in body weight (> 3 kg) over the previous 2 months. Participants were interviewed about their fish/seafood intake before they were included in the study, and those with a regular fish intake > 1 fish dinner per week were instructed to avoid eating fish for 4 weeks before the baseline visit. Very few participants (< 10%) had an intake above 1 fish dinner per week before enrolment to the study and went through a 4-week fish-free period before the start of the intervention period.

The study was designed as a randomised, controlled intervention study with a parallel group design, with three intervention arms: Atlantic cod (wild-caught *Gadus morhua*) in weekly doses of 750 g, Atlantic salmon (Norwegian farmed *Salmo salar*) in weekly doses of 750 g, and a no-fish group as the Control group. The intervention period was 8 weeks. In all, 76 participants were included in the study and were randomly assigned to the Cod group (*N* = 27), Salmon group (*N* = 27) or the Control group (*N* = 22). The participants were randomised into the different groups by the project manager by drawing lots, and the participants were informed about their group allocation during the baseline visit. All examinations were conducted at the Clinical Research Unit at the Haukeland University Hospital, Bergen, Norway. To enhance compliance, the participants were contacted by phone approximately 1 week prior to baseline and end point visits, during which they were informed of the schedule and procedures for the following visit. Also, a text message was sent 1–3 days before the 8 week visit, as a reminder of how to prepare for the upcoming visit. For any inquires during the trial period, members of the research group could be reached by email or telephone. Compliance was monitored through interviews; after 1, 4 and 8 weeks of the intervention period, the participants in the fish eating groups were asked how many dinners with cod/salmon they had not eaten since last contact, instead of asking how well they had complied, to make it less dissuasive for the participants to report missing intake. Noncompliance was defined as not following the protocol in regard to fish intake (omitting more than three fish dinners in the fish eating groups during the intervention period), other dietary changes or use of prescription medicine not compatible with the inclusion criteria, or changes in physical activity. As reward for completing the study, participants were offered a dietary consultation with a student dietician at the last visit and all the results of analyses of their blood samples.

The study was conducted according to the guidelines laid down in the Declaration of Helsinki, and all procedures were approved by the Regional Committee for Medical and Health Research Ethics of Western Norway (REC no.: 2011/572). Written informed consent was obtained from all subjects.

Health professionals performing blood sampling, and personnel conducting the laboratory analyses, were all blinded to participants’ identity and group allocation. All data were analysed anonymously. This trial was registered at clinicaltrials.gov as NCT02350595.

### Interventions

Cod and salmon fillets were provided to the participants as frozen-skin and boneless-fillet portions (mean weight with standard deviation; 150 (SD 10) g; Lerøy Seafood Group ASA). Pallets of fish were chosen at random from Lerøy’s warehouse in Bergen, Norway. The cod and salmon fillets were supplied free of charge to the participants, and were distributed at the baseline visit or at any time during the study period, if preferred. Participants in the Cod group were instructed to eat five dinners per week containing 150 g of cod fillet, and participants in the Salmon group were instructed to eat five dinners per week containing 150 g of salmon fillet. The participants in the fish-eating groups were told not to exceed a total amount of 750 g of fish/week, not to consume any other types of fish or seafood during the study period, and to maintain their normal eating habits throughout the study period apart from eating the mandatory amount of 750 g fish/week. The Control group was also instructed to continue their normal eating habits, except to avoid fish and seafood intake. Participants in both fish intervention groups received a booklet with dinner recipes for inspiration and to help them to increase the variation of their meals, as previously described [22]. The participants in the Control group did not receive any recipes, to avoid them being inspired to change their dietary habits during the study period.

Subjects in all groups were instructed not to change their physical activity level during the intervention period. The participants’ habitual lifestyle were recorded at the baseline and the endpoint visits, using a questionnaire for reporting physical activity. The participants were asked about the types of physical activity they engaged in, such as whether they worked out in a gym, were members of sports clubs or whether they worked out individually, the type of physical activity (e.g. hiking, running, biking) and the number of hours of light physical activity (not sweaty/not breathless) or hard physical activity (with sweat/breathless). The participants completed the questionnaire at the baseline and endpoint visits. The weekly number of hours and the intensities of the physical activities were coded as continuous variables, and reported physical activity were similar between the groups at baseline and were not changed within the groups during the study period [19].

### Protocol for study visits

The total study period was 8 weeks, with baseline visits between August 22, 2011 and September 19, 2011. Examinations were conducted in the morning after an overnight fast. The subjects were instructed not to eat or drink anything except water, and not to use substances containing nicotine after 22.00 h the previous day, and to avoid physical exercise and alcohol for 24 h before each sampling day.

Body height was measured at the baseline visit, using a wall-mounted stadiometer (Seca 222; Seca). Body weight and body composition were measured using a bioelectrical impedance analysis device (InBody 720; Biospace Co. Ltd) at the baseline and endpoint visits.

Fasting blood samples were collected at baseline and after 8 weeks, in BD Vacutainer SST II Advance gel tubes (Becton, Dickinson and Company) for isolation of serum. The staff complied with a strict protocol for pre-analytical sample handling to ensure high sample quality. Blood samples were centrifuged after 30 min at room temperature, and serum were immediately aliquoted and frozen at − 80 °C until analyses. Participants provided morning urine at both visits for measurements of markers of kidney function. Urine samples were immediately aliquoted and frozen at − 80 °C upon participants’ arrival to the hospital.

### Estimation of intakes of energy, macronutrients, iron, magnesium, selenium and zinc from dietary records

Participants completed dietary records of the 5 preceding days before the baseline visit and the 5 preceding days before the 8-week visit, including at least 1 weekend-day. The intakes of energy, protein, fat, saturated fatty acids (SFA), *cis*-monounsaturated fatty acids (*cis*-MUFA), *cis*-polyunsaturated fatty acids (*cis*-PUFA), carbohydrates, iron, magnesium, selenium and zinc were calculated from the participants’ dietary records using the ‘Mat på Data 5.1’ software [23], which contains information on the nutrient contents in food items sold in Norway. This food database does not contain information of copper contents in foods. Food records were checked for completeness at both study visits.

### Analyses of serum and urine samples

All serum/urine samples for each analysis were analysed pairwise for each participant in random order on the same day, and samples were not thawed previously. Total neopterin was measured in serum after precipitation of proteins by trichloroacetic acid, which oxidises 7,8-dihydroneopterin to neopterin, and total neopterin was analysed by Bevital AS (Bergen, Norway, http://www.bevital.no) using liquid chromatography combined with tandem mass spectrometry, as previously described [24]. Vitamin A (all-trans retinol) and vitamin E (α-tocopherol and γ-tocopherol) were measured by liquid chromatography-tandem mass spectrometry [25] by Bevital AS. Serum magnesium and iron were analysed with the Cobas c111 system (Roche Diagnostics GmbH, Marburg, Germany) using the MG (Magnesium) and IRON2 (Iron Gen.2) kits for Cobas c111 (Roche Diagnostics GmbH). Copper and selenium in serum were quantified by the Department of Medical Biochemistry and Pharmacology, Haukeland University Hospital, using an Elan DRC-e Inductively Coupled Plasma Mass Spectrometer (Perkin Elmer MDS Sciex, Concord, ON, Canada) in standard mode, after dilution 1:15 with 1% Triton^®^ X-100 and 0.33% w/v pro analysis HNO_3_.

Urine concentrations of cystatin C and T-cell immunoglobulin mucin-1 (TIM-1) were measured as markers of kidney function, using the Human Cystatin C Quantikine^®^ ELISA (DSCTC0) and the Human Urinary TIM-1/KIM-1/HAVCR Quantikine^®^ ELISA (DKM100) from R&D Systems (Bio-Techne). Creatinine in urine was quantified using the CREP2 (Creatinine plus ver.2) kit from Roche Diagnostics using the Cobas c111 system (Roche Diagnostics GmbH).

### Outcome measurements

The primary outcome of the present study was changes in serum total neopterin concentration after a weekly intake of 750 g fillet from either cod or salmon for 8 weeks. Secondary outcomes were changes in estimated dietary intakes of iron, magnesium, selenium and zinc, and changes in serum concentrations of copper, iron, magnesium, selenium, all-*trans* retinol and α-tocopherol.

### Sample size estimation

The sample size calculation for this trial was originally conducted with the aim to investigate the effects of high intake of cod or salmon on postprandial glucose regulation after a standardised breakfast in participants who were overweight or obese [19]. We have previously presented an estimate that it was necessary to include 76 participants divided into 3 groups to ensure that 20 participants in each group completed the trial with satisfactory compliance, with a power of 80% and *α* of 0.05, and of these, 65 participants were included in statistical analyses [19]. In the present study, we wanted to further explore serum and urine samples from this randomised clinical trial to investigate the effect of a high intake of cod or salmon on serum concentrations of total neopterin. This is the first study to investigate the effects of 8 weeks of high intake (750 g/week) of cod or salmon on serum total neopterin concentration in adults who were overweight or obese, therefore, no data are available for sample size calculation for the present study.

### Statistical analyses

Statistical analyses were conducted using SPSS Statistics 25 (SPSS, Inc., IBM Company). Subjects who did not complete the study were excluded from the statistical analyses. For analytes in serum and for estimated intake of micronutrients, most data were not normally distributed according to the Shapiro–Wilk test, therefore, the nonparametric Wilcoxon’s signed-ranks test was used to investigate changes within groups. The Kruskal–Wallis test was used to compare values between the three groups at baseline. Changes within the groups were compared using the Kruskal–Wallis test followed by group comparisons adjusted using the Bonferroni correction for multiple tests whenever between-group differences were detected. All statistical testing within and between the groups were unadjusted. Correlations between parameters were tested using two-tailed Spearman’s correlations test. Data are expressed as medians and 25th, 75th percentiles. Categorical data were compared using the Pearson’s *χ*^2^ test. All comparisons were two-sided, and *p* < 0.05 was considered statistically significant.

## Results

### Participant characteristics

**Fig. 1:**
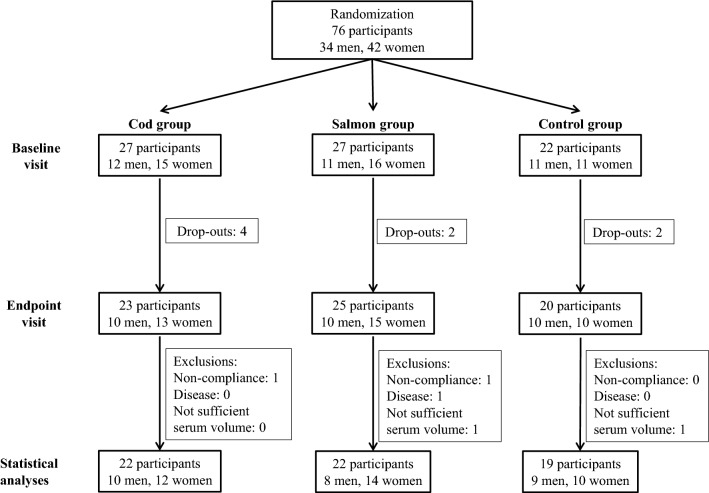
Study overview of participants. Participants who did not comply with the study protocol were excluded from statistical analysis. Noncompliance was defined as not following the protocol in regard to fish intake (omitting more than three fish dinners in the fish eating groups), other dietary changes or use of prescription medicine not compatible with the inclusion criteria, or changes in physical activity

In total, 76 participants were included in the study and completed the first study visit, and 68 participants completed the trial. One participant (a woman in the Salmon group) was excluded from statistical analysis after analyses of postprandial blood glucose revealed she had prediabetes, and two participants (one woman in the Cod group and one man in the Salmon group) were withdrawn from analysis because they did not comply with the protocol. From two of the participants (one man in the Salmon group and one man in the Control group), we did not have a sufficient amount of blood serum for analyses, and these two subjects were excluded from all analyses in the present paper. In total, 63 subjects (27 men and 36 women) were included in the statistical analyses. The flow of participants in the study is presented in Fig. 1.

Groups were similar at baseline in regard to gender distribution, age, BMI, percentage body fat or percentage muscle mass (Table 1), with median age 45.6 (25th,75th percentile 37.1,53.9) years and median BMI 32.3 (25th,75th percentile 29.6,35.7) kg/m^2^. After 8 weeks, no changes were seen in any of the groups for BMI or percentage body fat or muscle (data not presented).

**Table 1:** Participant characteristics at baseline (medians and 25th, 75th percentiles)

	Cod group (*N* = 22)		Salmon group (*N* = 22)		Control group (*N* = 19)		P*
Men/women	10/12		8/14		9/10		0.74
Age, years	47.2	38.0, 54.2	46.1	43.2, 52.6	40.1	31.0, 54.4	0.44
BMI, kg/m^2^	31.0	29.2, 35.9	32.2	29.9, 34.7	33.9	29.1, 36.6	0.73
Body fat, %	39.3	28.3, 42.9	40.2	30.1, 43.0	39.3	33.1, 41.2	0.92
Muscle mass, %	34.4	32.5, 41.4	33.3	31.5, 40.0	33.9	32.2, 38.2	0.72

Groups were compared at the baseline using the Pearson’s *χ*^2^ (categorical data) or the Kruskal–Wallis test (continuous data)

### Estimated dietary intakes of energy, macronutrients, iron, magnesium, selenium and zinc

The estimated median daily intakes of energy, protein, fat, SFA, cis-MUFA, cis-PUFA, carbohydrates, iron, magnesium, selenium and zinc were calculated from 5-day food diaries prior to baseline and endpoint visits, and intakes were similar between the groups at baseline. Participants reported no intake of seafood (beside the mandatory cod/salmon intake in the fish eating groups), liver or kidney in the food diaries. The selenium intake was similar in all groups, but tended to be increased in the Cod group (*P* = 0.052) and in the Salmon group (*P* = 0.086) from baseline to endpoint when compared to the Control group, with no difference between the Cod and the Salmon group (Table 2). The zinc intake was significantly reduced in the Cod group and unchanged in the Salmon group from baseline to endpoint when compared to the Control group, with no difference between the Cod group and the Salmon group. The intakes of energy, protein, fat, SFA, *cis*-MUFA, *cis*-PUFA, carbohydrates, iron and magnesium were not changed within any of the groups from baseline to 8 weeks.

**Table 2:** Estimated daily dietary intake of energy, macronutrients, iron, magnesium, selenium and zinc based on 5-day dietary records at baseline and after 8 weeks (medians and 25th, 75th percentiles)

	Baseline		8 weeks		*P*^†^	*P*^‡^	*P*^*§*^
	Median	25th, 75th percentile	Median	25th, 75th percentile			
Energy, kcal/day							
Cod group	1995	1776,2473	1963	1557,2372	0.41	0.44	
Salmon group	1974	1787,2422	1930	1821,2482	0.38		
Control group	2128	1873,2559	2120	1653,2790	0.50		
Protein, g/day							
Cod group	94	84,122	100	74,114	0.86	0.83	
Salmon group	89	77,105	92	83,98	0.85		
Control group	92	80,106	89	72,113	0.57		
Fat, g/day							
Cod group	90	68,115	83	66,108	0.45	0.17	
Salmon group	93	64,122	93	84,120	0.14		
Control group	87	73,114	91	80,117	0.058		
SFA, g/day							
Cod group	36	28,42	38	23,48	0.32	0.91	
Salmon group	31	25,51	35	30,44	0.43		
Control group	36	27,43	37	32,52	0.15		
*cis*-MUFA, g/day							
Cod group	33	23,40	29	20,36	0.43	0.17	
Salmon group	29	20,39	29	27,39	0.17		
Control group	30	23,43	32	30,42	0.058		
*cis*-PUFA, mg/day							
Cod group	16	10,23	12	9,19	0.21	0.36	
Salmon group	14	10,27	16	12,22	0.52		
Control group	14	10,19	13	11,17	0.93		
Carbohydrates, g/day							
Cod group	203	168,248	181	161,225	0.74	0.55	
Salmon group	196	162,232	188	144,233	0.18		
Control group	203	164,271	185	141,286	0.74		
Iron, mg/day							
Cod group	10.4	9.4,12.8	9.3	7.0,11.3	0.021	0.063	
Salmon group	10.7	8.8,12.1	9.4	7.7,11.4	0.27		
Control group	10.1	7.7,11.1	10.0	8.1,12.6	0.26		
Magnesium, mg/day							
Cod group	326	283,371	327	260,402	0.86	0.99	
Salmon group	319	298,362	327	278,369	0.95		
Control group	336	261,359	324	262,396	0.91		
Selenium, µg/day							
Cod group	60.5	47.8,74.0	75.0	65.5,91.0	0.044	0.033	0.052^A^
Salmon group	57.0	41.8,92.5	76.0	65.5,83.5	0.10		0.086^B^
Control group	52.0	36.0,72.0	47.0	33.5,54.8	0.45		1.00^C^
Zinc, mg/day							
Cod group	11.8	9.2,14.1	10.3	7.7,12.8	0.028	0.042	0.037^A^
Salmon group	10.2	8.9,12.4	8.8	7.2,11.6	0.24		0.31^B^
Control group	11.6	10.3,12.9	11.6	10.1,15.2	0.35		1.00^C^

No differences were seen between the groups at the baseline (Kruskal–Wallis test). Results are presented for 22 participants in the Cod group, 22 participants in the Salmon group and 19 participants in the Control group

^†^Within-group changes were tested using the Wilcoxon’s signed-ranks test

^‡^Changes within Cod group, Salmon group and Control group were compared using the Kruskal–Wallis test

^§^Changes within the Cod group were compared with the Control group (A), changes within the Salmon group were compared with the Control group (B), changes within the Cod group were compared with the Salmon group (C) when the Kruskal–Wallis test showed differences between the groups. P values for group comparisons were adjusted using the Bonferroni correction for multiple tests

### Concentrations of total neopterin and selected elements and vitamins in serum

**Fig. 2:**
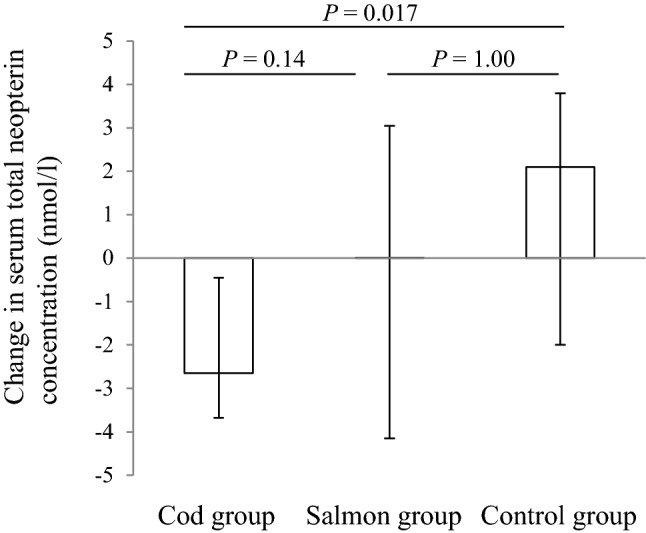
Within-group changes in serum concentrations of total neopterin from baseline to 8 weeks. Results are presented for 22 participants in the Cod group, 22 participants in the Salmon group and 19 participants in the Control group and are presented as medians and 25th, 75th percentiles. Changes within the Cod group, Salmon group and Control group were compared using the Kruskal–Wallis post hoc test for group comparisons. P values for group comparisons were adjusted using the Bonferroni correction for multiple tests

Serum concentrations of total neopterin, copper, iron, magnesium and selenium were similar between the groups at baseline. Total neopterin serum concentration was significantly reduced from baseline to endpoint in the Cod group when compared to the Control group, but this reduction was not significantly different when compared to the Salmon group, and the changes within the Salmon group and the Control group were not significantly different (Table 3, Fig. 2).

**Table 3:** Serum concentrations of total neopterin (7,8-dihydroneopterin + neopterin), copper, iron, magnesium and selenium at baseline and after 8 weeks (medians and 25th, 75th percentiles)

	Baseline		8 weeks		*P*^†^	*P*^‡^	*P*^§^
	Median	25th, 75th percentile	Median	25th, 75th percentile			
Total neopterin, nmol/l							
Cod group	21.5	19.0,25.8	19.1	15.8,22.3	0.0085	0.023	0.018^A^
Salmon group	21.4	16.3,24.9	19.7	18.5,25.3	0.79		0.59^B^
Control group	19.4	16.0,23.7	21.6	16.6,26.8	0.13		0.39^C^
Copper, µmol/l							
Cod group	17.2	14,4,18.6	16.1	13.9,18.9	0.70	0.34	
Salmon group	17.9	15.0,19.4	18.3	15.7,20.1	0.50		
Control group	17.1	16.4,19.6	17.2	16.0,19.3	0.083		
Iron, µmol/l							
Cod group	16.8	13.8,18.9	16.7	13.3,21.3	0.96	0.26	
Salmon group	14.7	10.9,20.1	13.9	11.4,18.1	0.24		
Control group	18.7	13.1,20.9	15.1	11.6,19.2	0.035		
Magnesium, mmol/l							
Cod group	0.86	0.83,0.92	0.85	0.80,0.88	0.0052	0.66	
Salmon group	0.86	0.82,0.89	0.85	0.82,0.88	0.22		
Control group	0.85	0.79,0.89	0.85	0.78,0.87	0.30		
Selenium, µmol/l							
Cod group	1.10	1.00,1.30	1.20	1.05,1.30	0.042	0.016	0.017^A^
Salmon group	1.15	1.08,1.23	1.10	1.10,1.20	0.87		1.00^B^
Control group	1.10	0.90,1.10	1.00	0.90,1.10	0.11		0.14^C^

No differences were seen between the groups at the baseline (Kruskal–Wallis test). Results are presented for 22 participants in the Cod group, 22 participants in the Salmon group and 19 participants in the Control group

^†^Within-group changes were tested using the Wilcoxon’s signed-ranks test

^‡^Changes within Cod group, Salmon group and Control group were compared using the Kruskal–Wallis test

^§^Changes within the Cod group were compared with the Control group (A), changes within the Salmon group were compared with the Control group (B), changes within the Cod group were compared with the Salmon group (C) when the Kruskal–Wallis test showed differences between the groups. *P* values for group comparisons were adjusted using the Bonferroni correction for multiple tests

The serum concentration of selenium was significantly increased in the Cod group when compared to the Control group, but was not significantly different from the Salmon group. The change in neopterin concentration from baseline to endpoint was similar in the Salmon group and the Control group. The magnesium serum concentration was reduced after 8 weeks in the Cod group, but this change was not significantly different from the changes in the Salmon group and the Control group. In the Control group, serum concentration of iron was reduced from baseline to endpoint, but this change was not significantly different from the fish eating groups. Serum concentrations of copper (Table 3) and all-*trans* retinol, α-tocopherol and γ-tocopherol [20] were not changed within or between the groups.

### Correlations between total neopterin serum concentration and other parameters

At baseline, serum concentration of total neopterin was not correlated with serum concentrations of copper, iron, magnesium, selenium, all-*trans* retinol, α-tocopherol and γ-tocopherol (Table 4). At endpoint, when all groups were considered together, serum total neopterin concentration was positively correlated with magnesium concentration (*r* = 0.368, *P* = 3.5 × 10^–3^), but was not correlated with the concentrations of the other elements or vitamins measured in serum (Table 5). When the experimental groups were investigated separately, no statistically significant correlations between serum total neopterin concentration and concentrations of elements and vitamins were seen within any of the groups (data not presented).

**Table 4:** Spearman’s correlations of serum concentrations of copper, iron, magnesium and selenium at baseline (*N* = 63)

	Neopterin^a^	Cu	Fe	Mg	Se	Vit A	aToc
Cu	− 0.202						
Fe	0.206	− 0.459**					
Mg	0.164	− 0.040	− 0.196				
Se	− 0.179	0.111	− 0.054	0.199			
Vit A	0.059	0.067	0.137	0.350**	0.563**		
aToc	0.057	0.136	− 0.053	0.371**	0.485**	0.405**	
gToc	0.124	0.005	− 0.008	0.150	− 0.090	0.067	0.289*

*Vit A* all-trans retinol, *aTOC* α-tocopherol, *gTOC* γ-tocopherol

^a^Total neopterin

*Correlation is significant at the 0.05 level (2-tailed)

** Correlation is significant at the 0.01 level (2-tailed)

**Table 5:** Spearman’s correlations of serum concentrations of copper, iron, magnesium and selenium at endpoint (*N* = 63)

	Neopterin^a^	Cu	Fe	Mg	Se	Vit A	aToc
Cu	− 0.177						
Fe	− 0.017	− 0.417**					
Mg	0.368**	− 0.208	− 0.179				
Se	− 0.185	− 0.041	0.211	0.127			
Vit A	− 0.113	− 0.024	0.174	0.198	0.563**		
aToc	− 0.152	0.068	0.101	0.244	0.483**	0.484**	
gToc	0.021	0.071	− 0.088	− 0.007	0.156	0.152	0.355**

*Vit A* all-trans retinol, *aTOC* α-tocopherol, *gTOC* γ-tocopherol

^a^Total neopterin

*Correlation is significant at the 0.05 level (two-tailed)

**Correlation is significant at the 0.01 level (two-tailed)

Cystatin C and TIM-1 were measured in urine sampled at baseline and endpoint as measures of kidney function since serum neopterin concentration is increased in patients with kidney dysfunction [26, 27]. We found no significant correlations between serum total neopterin concentration and urine concentrations (relative to creatinine) of cystatin C and TIM-1 at baseline and endpoint (*P* > 0.4, data not presented).

## Discussion

The effects of diets on serum neopterin concentration are largely unknown. In the present study, we observed that a weekly intake of 750 g Atlantic cod for 8 weeks reduced the serum concentration of total neopterin compared to the Control group. Total neopterin concentration provides a sensitive measure for cellular immune activation [4], and the reduced concentration after high cod intake suggests that immune activation was reduced. A high intake of Atlantic salmon (750 g/week) did not affect serum concentration of total neopterin, and the change in serum neopterin in this group was similar to that of the Cod group and the Control group. This is in line with our previous findings, where intake of lean fish (such as cod), but not of fatty fish (such as salmon) was inversely associated with circulating neopterin in an observational study in patients with coronary artery disease and median BMI of 26.4 kg/m^2^ [17].

The median serum total neopterin concentration for our overweight/obese participants at baseline was 21.1 (25th, 75th percentile 17.0, 24.2) nmol/l. This is marginally higher than the reported normal range for total neopterin in healthy normal-weight adults in serum that has not been previously thawed which is 17.3 nmol/l (geometric CV: 20%), with lower concentrations for each consecutive freeze/thaw cycle [28]. The serum samples used in the present study had not been thawed previously prior to analysis, and the high serum total neopterin concentration measured is a result of excellent pre-analytical sample handling resulting in higher neopterin concentrations than reported from studies where samples have been frozen and thawed multiple times. The biological variation in serum neopterin concentration at baseline was in part due to significantly higher serum neopterin concentration in men compared to women; median with 25th, 75th percentiles were 21.6 (18.3, 28.2) nmol/l for men and 19.7 (15.6, 23.4) nmol/l for women, with *P* = 0.013 for comparison of the sexes (Mann–Whitney test). In elderly adults without pre-existing coronary heart disease, a small reduction in neopterin concentration of only 1–2 nmol/l, especially in the lower concentration area, is associated with lower risk for acute coronary events [16]. Thus, although the reduction in serum total neopterin concentration in the Cod group was small (the median reduction was − 2.65 nmol/l, with 25th, 75th percentile − 3.68, − 0.45 nmol/l), this may be of potential clinical importance.

Persons with reduced kidney function have elevated circulating concentration of neopterin [26, 27]; however, all participants in the present study had apparently normal kidney function based on measurements of serum creatinine and urine albumin [29]. In addition, since we found no correlations between serum total neopterin and markers cystatin C and TIM-1 in urine, both considered to be useful early indicators of renal damage [30, 31], this implies that the observed change in serum total neopterin concentration in the Cod group was not caused by changes in renal function.

The production of neopterin in macrophages is stimulated by IFNγ during immune activation [4], but IFNγ also triggers production of reactive oxygen species in macrophages [5]. Thus, neopterin may be an indicator of oxidative stress accompanying immune activation [6], and findings by Murr et al. shows that neopterin concentration is inversely correlated with the antioxidant enzyme cofactor selenium [7] and antioxidant vitamins such as α-tocopherol [8] in circulation. Not only selenium but also copper, iron, magnesium and zinc are essential for the activity of antioxidant enzymes and may counteract and prevent damage caused by free radicals; however, we found no correlations between the serum concentrations of these elements or all-trans retinol, α-tocopherol and γ-tocopherol with serum total neopterin concentration at baseline in our study. In the endpoint dataset, serum total neopterin was positively correlated with serum magnesium concentration but not with the measured trace elements or antioxidant fat-soluble vitamins. Our findings are in contrast with findings by Murr et al. showing inverse correlation of neopterin concentration with selenium [7] and α-tocopherol [8] in patients with cardiac disorders, possibly due to the larger concentration span of neopterin in their patients compared to our healthy participants with overweight/obesity. The lack of correlation between serum total neopterin concentration and concentrations of selenium, all-*trans* retinol, α-tocopherol and γ-tocopherol in our data set thus weaken the argument that there is a causal effect of selenium and fat-soluble antioxidant vitamins on total neopterin concentration.

Fillets from fish and organ meats such as liver and kidneys are particularly rich in selenium. None of the participants in any of the three groups reported intake of liver or kidney in their food diaries in the 5-day periods before the baseline or endpoint visits [20]. Our participants in the fish eating groups consumed high amounts of cod or salmon, which are good dietary sources for selenium with comparable content of selenium (30 µg Se/100 g fillet for both [23]). The estimated daily selenium intake based on the food diaries was similar in all groups at endpoint, but tended to be higher in both fish eating groups compared to the Control group. The serum selenium concentration was increased in the Cod group and not in the Salmon group when compared to the Control group, but both fish eating groups experienced a similar change in serum selenium concentration during the intervention period.

We have previously shown that the contents of long chain n-3 *cis*-PUFAs (EPA, DHA, DPA) in leucocyte membranes were significantly higher in participants in the Salmon group compared to those in the Cod group and the Control group [19], as a result of the high n-3 content in salmon. Unfortunately, we could not estimate the dietary intake of n-3 *cis*-PUFAs since the food database ‘Mat på data’ does not distinguish between n-3 and n-6 *cis*-PUFAs in food items. The n-3 PUFAs are more prone to oxidation by reactive oxygen species since they have a higher number of double bonds when compared to n-6 PUFAs with similar chain length, thus potentially increasing the level of oxidative stress after high salmon intake. In addition, the content of the antioxidant taurine [32] is two times higher in cod fillet compared to salmon fillet [33] and may contribute to an antioxidant effect of cod intake. Thus, the oxidative stress may be lower in the Cod group compared to the Salmon group. This is further supported by findings showing lower oxidative stress and increased plasma antioxidant capacity in adults consuming 3 × 150 g weekly portions of cod, with no effect of the corresponding amounts of salmon, as part of calorie-restricted diets [34].

This study has some strengths and limitations. Strengths include the excellent pre-analytical sample handling and the sensitive method for total neopterin quantification, and the measurement of several elements that are essential for the activity of antioxidant enzymes in addition to fat-soluble antioxidant vitamins. A limitation is the generalisability for the present findings, since the fish intake was very high in the Cod and Salmon groups, only two types of fish were used in the intervention diets, and the study population was relatively small and consisted only of adults who were overweight or obese. The results obtained may not be valid for individuals with different demographics including BMI and different lifestyles than the participants in the present study. Therefore, additional studies are needed to address these factors. The cod and salmon fillets used in the present study were randomly chosen from different batches in Lerøy’s warehouse in Bergen, and were representative for cod and salmon sold in Norwegian grocery stores. We did not conduct our own analyses of minerals, trace elements or vitamin contents in these batches, but instead we used average values from the official Norwegian and Swedish food databases. The present study is considered to be a pilot study that is hypothesis generating rather than hypothesis testing, as there is no information available about the necessary sample size for investigating the effects of high fish intake on serum neopterin concentration. Therefore, this study will constitute a base for sample size calculations for future studies with similar designs.

To conclude, the results from this study show that a weekly intake of 750 g wild-caught Atlantic cod for 8 weeks reduced the serum concentration of total neopterin when compared to a control group that did not consume fish or seafood. Intake of farmed Atlantic salmon (750 g/week) did not affect serum total neopterin concentration. The observed reduction in serum total neopterin concentration after cod intake was small; the median reduction was − 2.65 (25th, 75th percentile − 3.68, − 0.45) nmol/l. Still, this may be of potential clinical importance since a small reduction in neopterin of only 1–2 nmol/l, especially in the lower concentration area, has been associated with reduced risk for acute coronary events in elderly adults without pre-existing coronary heart disease [16].
